# Balanced Genome Triplication in Wheat Causes Premature Growth Arrest and an Upheaval of Genome-Wide Gene Regulation

**DOI:** 10.3389/fgene.2020.00687

**Published:** 2020-07-08

**Authors:** Xiaowan Gou, Ruili Lv, Changyi Wang, Tiansi Fu, Yan Sha, Lei Gong, Huakun Zhang, Bao Liu

**Affiliations:** ^1^School of Life Sciences, Jiangsu Normal University, Xuzhou, China; ^2^Key Laboratory of Molecular Epigenetics, Northeast Normal University, Changchun, China

**Keywords:** ploidy level, genome multiplication, transcriptome shock, dysregulation, ploidy syndrome, *Triticum aestivum*

## Abstract

Polyploidy, or whole genome duplication (WGD), is a driving evolutionary force across the tree of life and has played a pervasive role in the evolution of the plant kingdom. It is generally believed that a major genetic attribute contributing to the success of polyploidy is increased gene and genome dosage. The evolution of polyploid wheat has lent support to this scenario. Wheat has evolved at three ploidal levels: diploidy, tetraploidy, and hexaploidy. Ample evidence testifies that the evolutionary success, be it with respect to evolvability, natural adaptability, or domestication has dramatically increased with each elevation of the ploidal levels. A long-standing question is what would be the outcome if a further elevation of ploidy is superimposed on hexaploid wheat? Here, we characterized a spontaneously occurring nonaploid wheat individual in selfed progenies of synthetic hexaploid wheat and compared it with its isogenic hexaploid siblings at the phenotypic, cytological, and genome-wide gene-expression levels. The nonaploid manifested severe defects in growth and development, albeit with a balanced triplication of the three wheat subgenomes. Transcriptomic profiling of the second leaf of nonaploid, taken at a stage when phenotypic abnormality was not yet discernible, already revealed significant dysregulation in global-scale gene expression with *ca.* 25.2% of the 49,436 expressed genes being differentially expressed genes (DEGs) at a twofold change cutoff relative to the hexaploid counterpart. Both up- and downregulated DEGs were identified in the nonaploid vs. hexaploid, including 457 genes showing qualitative alteration, i.e., silencing or activation. Impaired functionality at both cellular and organismal levels was inferred from gene ontology analysis of the DEGs. Homoeologous expression analysis of 9,574 sets of syntenic triads indicated that, compared with hexaploid, the proportions showing various homeologous expression patterns were highly conserved in the nonaploid although gene identity showed moderate reshuffling among some of the patterns in the nonaploid. Together, our results suggest hexaploidy is likely the upper limit of ploidy level in wheat; crossing this threshold incurs severe ploidy syndrome that is preceded by disruptive dysregulation of global gene expression.

## Introduction

Polyploidy, or whole-genome duplication (WGD), is an important evolutionary force that has shaped the genomes of all higher plants and many animals (Van de Peer et al., 2009; Jiao et al., 2011; Paterson et al., 2012; Soltis and Soltis, 2012; Jiao and Paterson, 2014; Wendel, 2015; Schwager et al., 2017; Van de Peer et al., 2017; Blischak et al., 2018; Spoelhof et al., 2019). Apart from evolutionary importance, polyploidy also bears significant contemporary relevance to human health (Gjelsvik et al., 2019), and crop improvement and is becoming increasingly topical in diverse research fields. Myriad attributes characterize polyploidy relative to its diploid counterpart with genome-wide increase of DNA content and gene dosage being at the root. Interspecific hybridization is another major evolutionary force that facilitates adaptation and speciation primarily via new combinations of old parental genetic variants (Soltis and Soltis, 2009; Marques et al., 2019), but it may also include immediate effects of genome shock (McClintock, 1984) that induce new genetic and epigenetic variations (Kashkush et al., 2002; Senerchia et al., 2015). Interspecific hybridization either occurs at the homoploid level or is coupled with WGD, i.e., allopolyploidization, with the latter being particularly prevalent in plants. The combined effects of genome merger and WGD may cause a stronger genome shock and are more potent in the generation of heritable variations than WGD alone as evidenced by a large body of empirical studies (Adams, 2007; Doyle et al., 2008; Feldman and Levy, 2012; Madlung and Wendel, 2013; Yoo et al., 2014; Song and Chen, 2015; Wendel, 2015). Consequently, polyploidy in general and allopolyploidy in particular are often associated with enhanced organismal performance in growth, yield, fitness, and evolvability (Comai, 2005; Doyle et al., 2008; Wendel, 2015; Van de Peer et al., 2017).

Apart from genetic consequences of WGD, many aspects of physiology are instantaneously affected in polyploids, which contribute to their altered metabolism and phenotypes, such as nuclear volume; cell size and number; and organ/organism structure, shape, and size. In fact, every aspect of cell activity can be affected in a polyploid due to physiological alterations (Doyle and Coate, 2019) because different cellular components may not scale proportionally with elevated ploidy level (Storchova et al., 2006). Thus, altered physiology alone without invoking genetic properties may constitute a constraint limiting the extent to which the number of genome multiplications can be tolerated by a given organism. Notably, while the extent of genome multiplication in certain terminally differentiated tissues/organs does not appear to be under strict control (Doyle and Coate, 2019), it apparently does at the organismal level. Indeed, several studies have shown that there exists an upper limit for the number of genome multiplications at the organismal level for a given species. For instance, Corneillie et al. (2019) assessed growth rates, biomass, and cell wall composition in *Arabidopsis thaliana* autopolyploid plants of three ploidal levels (2*n* = 4*x*, 6*x*, and 8*x*) relative to their isogenic diploid (2*x*) counterpart and found that only tetraploid exhibited superior performance in all the analyzed traits, while hexaploid and octaploid plants did not show advantages in all traits or even manifested apparent defects in most traits in the octaploid. This is consistent with earlier findings in *A. thaliana* by Tsukaya (2008), who described the phenomenon “high ploidy syndrome,” i.e., higher ploidy level often exhibits retarded growth and trade-offs between cellular and organ size or organismal size. The detrimental effects on growth by higher ploidy are thought to be due to burdens on cell cycle and, hence, reduced cell division rate as a result of increased DNA content (Tsukaya, 2008).

Conceivably, interplays exist between the physiological effects and genetic consequences of genome multiplication. For example, disproportional scaling in surface area and length of spindle pole body generates geometric constraints that results in chromosomal instability in yeast (Storchova et al., 2006). Also, genome-wide changes in gene expression are expected to be an automatic outcome, i.e., transcriptomic response, as a result of a genome-wide increase of gene dosage. Although gene expression analyses associated with polyploidization have been mainly conducted in allopolyploids in which the combined effects of hybridization and WGD are involved (Doyle et al., 2008; Yoo et al., 2014; Song and Chen, 2015), several recent studies have focused on autopolyploids, i.e., the pure effect of genome multiplication. It was found that WGD *per se* alters expression of a substantial proportion of the transcriptome, especially when changes in transcriptome size was taken into account (Robinson et al., 2018; Doyle and Coate, 2019; Spoelhof et al., 2019; Visger et al., 2019). Hitherto, a relationship between transcriptomic response and “high ploidy syndrome” (Tsukaya, 2008) remains poorly understood.

The wheat genus (*Triticum*) comprises species at three ploidal levels (2*x*, 4*x*, and 6*x*), and remarkably, at least one species at each ploidal level has been successfully domesticated into major food crops (Matsuoka, 2011; Feldman and Levy, 2015). Nevertheless, wheat species of the three ploidal levels differ dramatically with respect to both their evolvability under natural conditions, for example, differential niche expansion ranges between wild diploid and tetraploid wheat (Salamini et al., 2002), and their human-mediated dispersions, which are dramatically different among the domesticated diploid, tetraploid, and hexaploid wheat (Dubcovsky and Dvorak, 2007). In fact, the emergence of each domesticated wheat of a higher ploidy (tetraploid vs. diploid and hexaploid vs. tetraploid) has rapidly replaced the lower ploidy one. This suggests superiority of increased ploidal level in the wheat species as allopolyploids. A long-standing issue, which to our knowledge has not been addressed, is what would be the outcome if a further whole genome multiplication is superimposed on hexaploid wheat?

Here, we characterize a spontaneously occurring nonaploid wheat individual in selfed progenies of a synthetic hexaploid wheat and compared it with its isogenic hexaploid siblings at the phenotypic, cytological, and genome-wide gene-expression levels. Our results suggest triplication of the three subgenomes of hexaploid wheat, let alone expected problems in meiosis as anisoploidy already imposes a severe detrimental effect on global gene regulation and growth/development at the vegetative stages, suggesting hexaploidy is likely the upper limit of ploidal level in wheat.

## Materials and Methods

### Plant Materials

The seeds of allohexaploid wheat line AT5 at the S_0_ generation along with its parental lines were procured from Moshe Feldman (Weizmann Institute of Science, Israel). This line was produced by hybridization of *Triticum turgidum* ssp. *durum* (accession TTR04) and *Aegilops tauschii* (accession TQ27), followed by colchicine treatment to induce chromosome doubling. All plant individuals were propagated under strict selfing conditions to produce advanced generations of AT5 from S_0_ to S_6_. All plants were grown in a common condition (day/night, 25°C/16°C, 16 h/8 h). When the third leaf appeared, and the second leaf fully expanded (Simmons et al., 1985), the second leaf of hexaploidy and nonaploidy was collected for RNA isolation. All collected leaves were kept at −80°C until use.

### Karyotyping by Sequential Fluorescence *in situ* Hybridization and Genomic *in situ* Hybridization

The protocols for fluorescence *in situ* hybridization (FISH) and genomic *in situ* hybridization (GISH) were essentially as described in Kato et al. (2004) with minor modifications. For FISH, two repetitive DNA sequences (pSc119.2 and pAS1) were labeled by nick translation with Alexa Fluor 488-5-dUTP (green) and Texas Red-5-dCTP (red), respectively. For GISH, genomic DNA of *T. urartu* and *Ae. tauschii* was labeled with Alexa Fluor 488-5-dUTP (green coloration), and Texas Red-5-dCTP (red coloration), respectively. Genomic DNA of *Ae. speltoides* was used as a blocker.

Metaphase chromosome spreads were prepared following Kato et al. (2004). Slide denaturation, hybridization, and washing conditions were carried out following the manufacturer’s recommendations (Invitrogen; no. C11397). Slides were examined with an Olympus BX61 fluorescence microscope and digitally photographed. The images were captured using the Olympus IPP software package and visualized in Photoshop CS 6.0 version.

### RNA-Seq Data Processing and Analysis

Total RNAs were isolated from the leaves using Trizol reagent (Invitrogen, United States) according to the manufacturer’s instructions. The integrity, quality, and concentration of extracted RNAs were assessed with the Agilent 2100 Bioanalyzer (Agilent Technologies, United States). Transcriptome libraries were constructed for each sample and sequenced using the Illumina HiSeq 2000 platform with standard protocols. Three biological replications were used for the hexaploid plants, and three technical replications were used for the nonaploid plant.

The hexaploid wheat (Chinese Spring) reference genome sequence and its annotation information were downloaded from IWGSC^1^. Each set of cleaned data was aligned to the reference using HISAT2 (version 2.0.1; Kim et al., 2015). The clean data information and mapping rates are shown in Supplementary Table S1. The uniquely mapped reads to the reference sequence were computed. The differentially expressed genes (DEGs) were determined by using Cuffdiff (version 2.2.1) by comparing the FPKM values. Transcripts with an FDR-adjusted (Benjamini and Hochberg, 1995) *p* < 0.05 and fold change > 2 were considered to exhibit statistically significant expression differences between samples.

We utilized triad genes that had a 1:1:1 correspondence across the three homoeologous subgenomes in hexaploid common wheat, the definition of homoeolog expression patterns was as reported (Ramirez-Gonzalez et al., 2018). We compared the seven homoeolog expression bias categories in the nonaploid relative to those in the hexaploid wheat.

Gene ontology (GO) enrichment analysis was performed by hypergeometric distribution in R (version 3.4.0) with an adjusted *p* < 0.05 as a cutoff to determine significantly enriched GO terms.

### Pyrosequencing

The protocol essentially followed the original report (Mochida et al., 2003) with modifications (Zhang et al., 2013). A set of balanced expressed triads were arbitrarily selected to design the pyrosequencing primers for the purpose of assaying subgenome-specific expression by the pyrosequencing system (PyroMarkID Q96, Qiagen, Germany). The SeqMan program^2^ was used to identify subgenome-specific single-nucleotide polymorphisms (SNPs) that enable reliable distinction of the A, B, and D subgenomes for a given triad gene. Consequently, both pyrosequencing primers and gene-specific PCR amplification primers were designed successfully for a set of 18 triads using the Soft Assay Design software. Biotin-labeled PCR products were immobilized on streptavidin-coated paramagnetic beads. Capture of biotinylated single-strand PCR products, annealing of the sequencing primer, and solid-phase pyrosequencing were performed following the manufacturer’s recommendations.

## Results

### The Nonaploid Wheat Contained a Balanced Genome Constitution Yet Manifested Severe Organismal Abnormality in Growth and Development

**FIGURE 1:**
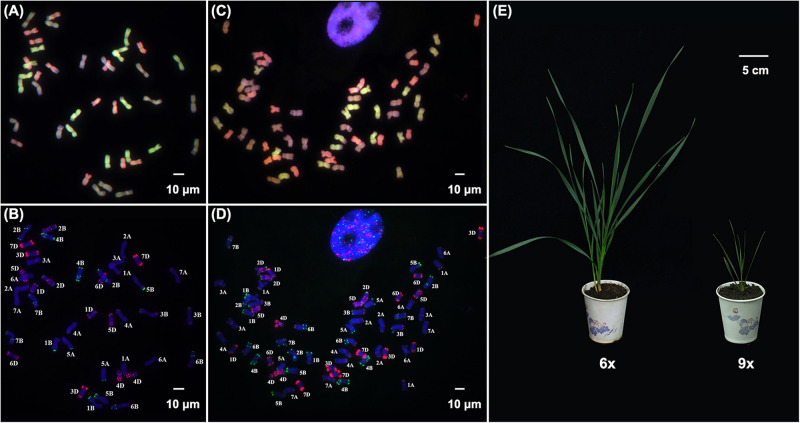
Karyotype and phenotype in hexaploid and nonaploid wheat. **(A)** and **(B)**, FISH and GISH images of a metaphase cell of hexaploid wheat. **(C)** and **(D)**, FISH and GISH images of a metaphase cell of nonaploid wheat. **(E)** Seedlings of hexaploid and nonaploid wheat. The FISH probes used are pSc119.2 (green), and pAS1 (red), the GISH probes used are genomic DNA from *Triticum urartu* (genome AA, green), and *Aegilops tauchii* (genome DD, red); and genome BB (blue) was counterstained with DAPI.

We used a combinatorial FISH and GISH procedure that allows unequivocal identification of each of the 21 wheat chromosome pairs (Zhang et al., 2013) to study chromosomal stability in various types of newly synthesized wheat. We analyzed a fifth-selfed generation (S_5_) plant population that originated from an individual plant of a synthetic allohexaploid wheat (dubbed AT5, 2*n* = 6*x* = 42, BBAADD) formed by crossing a durum wheat (*T. turgidum*, ssp. *durum*, cv. TTR04, 2*n* = 4*x* = 28, BBAA) and *Aegilops tauchii* (accession TQ27, 2*n* = 2*x* = 14, DD), followed by colchicine-induced chromosome doubling. In the course, we identified a spontaneously formed nonaploid plant among 350 karyotyped individuals. This nonaploid plant contained three complete sets of the A, B, and D subgenomes (2*n* = 9*x* = 63, BBBAAADDD) of hexaploid common wheat with no evidence of numerical or structural chromosomal abnormality across all the 15 root-tip cells examined (Figures 1A–D). This suggests the nonaploid plant resulted from a fusion of an unreduced 2n gamete (6*x*) with a normal 1n gamete (3*x*) and with normal mitotic cell divisions. The individual nonaploid seed was not recognized because it appeared normal and showed no difference from those of the hexaploids, which also germinated regularly. However, compared with its isogenic hexaploid siblings, the germinated nonaploid seedling plant manifested conspicuous retardation in growth and development soon after the second-leaf stage with fewer leaves and a single tiller 45 days postgermination (Figure 1E). Moreover, it did not show further growth henceforth, failed to enter the reproductive stage, and died around 60 days postgermination.

### Massive Global Dysregulation of Gene Expression in Nonaploid Wheat

The impact of genome multiplication *per se* on gene expression in plants remains controversial and tends to be case-specific (Spoelhof et al., 2019). To test whether the abnormal growth and development of the nonaploid wheat were associated with major changes in gene expression due to genome triplication, we conducted a deep RNA-seq-based transcriptome analysis. We used the fully expanded seconf leaf (Simmons et al., 1985) as the target tissue because at this developmental phase no discernible difference between seedlings and the leaves was seen between the nonaploid and its isogenic euploid hexaploid siblings although abnormality in the nonaploid appeared henceforth. Assessing the obtained RNA-seq data indicated that reads of the single nonaploid plant exceeded 109 million while those of the hexaploid plants (with three biological replications) reached up to 188 million. Nevertheless, this difference in sequence depth should not impede reliable comparative analysis because 109 million was already equivalent to 30x coverage of a haploid genome for hexaploid wheat. The reads of each sample were mapped to the updated version of the hexaploid common wheat (cv. CS) reference genome (International Wheat Genome Sequencing Constorium et al., 2018) with an average mapping rate of *ca.* 90%. Correlation coefficients across the three biological replicates of the hexaploid plants were 0.96 ∼ 0.98 (Supplementary Table S1), indicating high robustness of our RNA-seq data and analyses.

**FIGURE 2:**
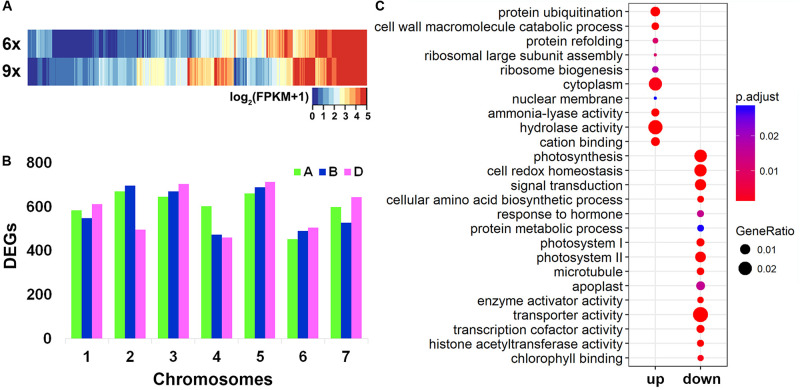
Distribution of differentially expressed genes (DEGs) in chromosomes and gene ontology (GO) analysis. **(A)** A heat map of DEGs according to the FPKM values between hexaploid and nonaploid wheat. **(B)** The distribution of DEGs in the 21 wheat chromosome pairs. **(C)** Enriched GO by up- and downregulated DEGs, respectively.

Collectively, 49,436 genes were found to be expressed in the hexaploid and nonaploid leaf samples (Table 1). We used the Cuffdiff software to quantify the DEGs between the nonaploid and hexaploid samples. Surprisingly, we found that, at a twofold change cutoff, 12,454 genes (25.2% of 49,436) were differentially expressed in the nonaploid leaf tissue compared with that of its isogenic hexaploid siblings. Of these DEGs, 7,998 (64.2%), and 4,456 (35.8%) were up- and down-regulated, respectively, in the nonaploid (Table 1 and Figure 2A) with the former being significantly more than the later (prop test, *p* < 2.2e–16). The DEGs were more or less evenly distributed among the 21 chromosome pairs, indicating that balanced triplication of the wheat genome resulted in large-scale global changes of gene expression (Figure 2B).

**TABLE 1:** The numbers of total expressed genes (EGs) and differentially expressed genes (DEGs) in nonaploid vs. hexaploid wheat plants.

Sample	EGs	DEGs*	Upregulated	Downregulated
9x vs. 6x	49,436	12,454	7,998	4,456

DEG*: fold-change > 2.

Notably, among the DEGs, 357 genes were non-expressed in hexaploid but activated in nonaploid, and 100 genes expressed in hexaploid were silenced in nonaploid (Supplementary Table S3), further pointing to a largely disruptive effect of genome triplication on transcriptional gene regulation.

To test whether specific cellular or organismal functions might have been impacted by the massive changes of gene expression in general and the nonaploid vs. hexaploid activated or silenced genes in particular, we conducted GO analyses for the various groups of DEGs. We found the up- and down-regulated DEGs, each as a group, showed distinct GO enrichments. The quantitatively upregulated DEGs were mainly enriched in cellular functions related to post-transcriptional regulation, including cytoplasm function, ribosome biogenesis, protein refolding and ubiquitination, and hydrolase activity, and the enriched GO terms for the down-regulated DEGs were mainly involved in organismal growth and development, including photosynthesis, signal transduction, response to hormones (Figure 2C). Notably, although the 357 nonaploid vs. hexaploid activated genes did not show significant enrichment, the 100 nonaploid vs. hexaploid silenced genes were overrepresented by GO terms involved in photosynthesis and auxin-response pathways, suggesting the qualitative misregulation of these genes (shutdown) may have played a major part in the impaired growth and development arrest of the nonaploid plant (Supplementary Figure S1).

### Only Moderate Perturbation of Relative Subgenome Homeologous Expression Patterns in the Nonaploid

It has been established that gene expression of the three homoeologous subgenomes of hexaploid wheat is highly coordinated (Ramirez-Gonzalez et al., 2018). To explore whether balanced triplication of the three subgenomes would disrupt the inherent subgenome expression patterns established in hexaploid wheat, we analyzed the triad genes that had a 1:1:1 correspondence across the three subgenomes in hexaploid wheat (Ramirez-Gonzalez et al., 2018). In the wheat cv. Chinese Spring (CS) reference genome, there were 17,753 sets of expressed triads (i.e., 53,259 genes) in total, including 1,007 non-syntenic triad sets (Ramirez-Gonzalez et al., 2018). We focused on the 9,574 sets of syntenic triads only because we found they were expressed in leaf tissue of both our nonaploid and hexaploid plants. The fragments per kilobase of exon model per million mapped fragments (FPKM) values of these genes were used for cluster analysis. We found that the subgenomes of both nonaploid and hexaploid were preferentially clustered together based on overall subgenome-specific expression level (relative subgenome transcript abundance) similarities (Supplementary Figure S2), suggesting balanced genome triplication did not cause a general disruption of the evolved homoeologous expression patterns (subgenome partitioning) of hexaploid wheat.

**FIGURE 3:**
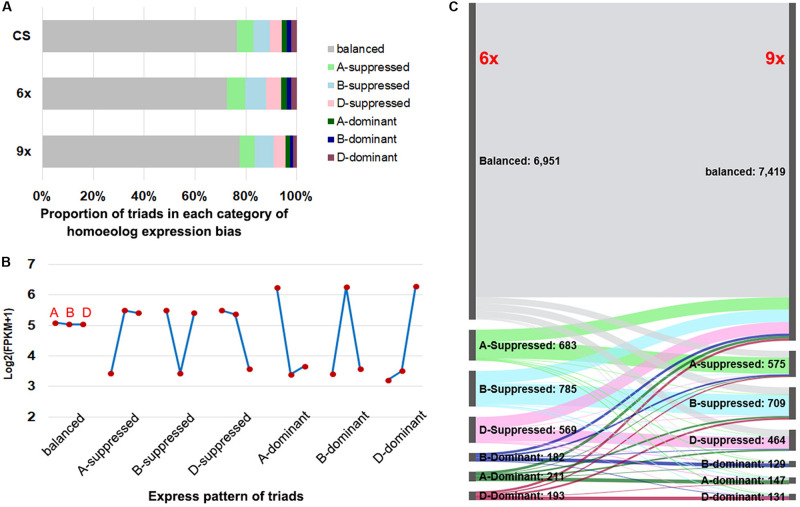
The expression pattern of triad genes between hexaploid and nonaploid wheat. **(A)** Proportion of triads in seven categories of homeolog expression bias in CS, hexaploid and nonaploid plants. **(B)** The model of average FPKM values for each subgenome from seven categories. **(C)** Alluvial plot of classification of triads between hexaploid and nonaploid wheat.

According to the prior established classification criteria (Ramirez-Gonzalez et al., 2018), we divided the triad genes into two major categories: balanced, i.e., those with a similar (statistically equal) relative abundance of transcripts from the three subgenome homeologs, and unbalanced, which were further divided into six subcategories, including homeolog dominant or suppressed, depending on the relative higher or lower abundance of transcripts from a single homeolog relative to those of the other two (Figure 3B and Supplementary Figure S3). Of the total 9,574 sets of expressed triads, the balanced category occupied the predominant proportions in both nonaploid and hexaploid, which were 7,419 (77.5%) and 6,951 (72.6%), respectively; the proportions of the single-homeolog dominant expression pattern were of the lowest category in both nonaploid (4.3%) and hexaploid (6.1%), and proportions of the single-homeolog suppressed category were in between for both nonaploid (18.3%) and hexaploid (21.3%). These proportions of triad expression patterns were also very similar to those seen in the same tissue of CS plants grown under our normal condition (Figure 3A). These results indicate that the proportions of triads manifesting each type of homoeologous expression patterns are highly conserved between the nonaploid and hexaploid wheat.

Nevertheless, conservation in proportion does not equate to the conservation of gene content or identity in each of the triad gene expression patterns between the nonaploid and hexaploid. To resolve this issue, we traced the expression pattern for each of the 7,775 sets of triads from hexaploid to nonaploid. We found homoeologous expression patterns in 81.2% (7,775 out of 9,574) of the triads remained the same between hexaploid and nonaploid while those in the remaining 18.8% (1,799 out of 9,574) triads changed to different patterns (Figure 3C). Of note, the pattern-changed triads mainly concerned the suppressed and dominant categories, which shifted to the balanced category or vice versa with few triads manifesting shifting to direct opposite patterns, i.e., from suppressed to dominant or vice versa (Figure 3C).

Taken together, it seemed that balanced genome triplication in wheat did not invoke a major perturbation to the highly coordinated subgenomic or homeologous expression patterns in hexaploid wheat (Ramirez-Gonzalez et al., 2018). To experimentally test the reliability of our subgenome expression patterns as well as of our RNA-seq analyses in general, we performed locus-specific cDNA pyrosequencing for a subset of 18 triads that belonged to the balanced category, which harbored diagnostic SNPs that enabled the design of pyrosequencing primers (Supplementary Table S2). For each of the 18 triads, the ratio of transcripts from the three subgenomes was determined. We found that in all 18 triads the pyrosequencing data were in line with the RNA-seq data (Supplementary Figure S4).

## Discussion

In this study, we compared a spontaneously formed nonaploid wheat individual with its isogenic hexaploid siblings at multiple levels, karyotype, phenotype, and gene expression. We found that the nonaploid individual maintained a stable somatic karyotype with no numerical or structural chromosomal variations detected in any of the 15 well-spread root-tip metaphase cells examined, suggesting mitosis was not impaired in the nonaploid with a balanced genome triplication. However, the nonaploid showed severe growth retardation and overall morphological abnormality immediately after the second-leaf stage, leading to premature death. In parallel, large-scale alteration of transcriptional gene expression was detected in the nonaploid relative to its isogenic hexaploid siblings in leaf tissue before the manifestation of phenotypic abnormality, suggesting gene dysregulation precedes the phenotypic abnormality. Due to limitation of material from the single nonaploid plant, we could not conduct experiments assessing possible differences in transcriptome sizes (Doyle and Coate, 2019) between the isogenic nonaploid and hexaploid plants. However, according to recent studies (Spoelhof et al., 2019; Visger et al., 2019), transcriptome size can be significantly altered due to an increase in ploidal level, and when this factor was taken into account, a substantially greater number of DEGs were identified (Visger et al., 2019). Thus, it is reasonable to deduce that the number of DEGs we identified in the nonaploid vs. hexaploid comparisons is an underestimate.

Notably, albeit there was upheaval of the overall gene expression level in the nonaploid, the tightly controlled inter-subgenome relative expression in hexaploid wheat (Ramirez-Gonzalez et al., 2018) was largely maintained. This is in contrast with the situation of aneuploidy in hexaploid wheat in which numerical change of a single chromosome caused large perturbation of subgenome expression (Zhang et al., 2017), suggesting coordinated subgenome regulation is determined by genome balance rather than genome dosage (Birchler and Veitia, 2012). This upheaval of overall gene expression vs. largely stable subgenome relative expression is also consistent with the possibility that changes in epigenetic regulation have played a role. Chromatin epigenetic modifications, including DNA methylation, histone modification and differential titration of regulatory small RNAs, are known to undergo variations due to polyploidization (Song and Chen, 2015). Given that epigenetic modifications are brought about by specific enzymatic machinery that acts *in trans* (Springer et al., 2016), it is conceivable that, in cases of allopolyploidy, they would affect all constituent subgenomes and, hence, cause alterations in overall gene expression rather than in subgenome-specific or -preferred expression.

Due to severe retardation of growth/development and inviability of the single nonaploid plant, we were also unable to perform more intricate analyses, such as measuring cell size/number, cell division rate, or cell wall composition as was done in the *A. thaliana* ploidal series (Corneillie et al., 2019). However, it is conceivable that perturbation of similar physiological and cell biological attributes typical of high ploidy syndrome (Tsukaya, 2008) might have been incurred in the nonaploid wheat although, in our case, the syndrome is apparently more drastic as it culminates in premature lethality. We can rule out disrupted mitosis as a cause for the syndrome (Comai, 2005) as all examined metaphase cells are euploidy, and also, no lagging chromosome was detected at anaphase. Thus, the ploidy syndrome in our case is most probably due to disruption of overall gene regulation, which affected a substantial proportion of the expressed genes, including activation and silencing of critical genes involved in both fundamental cellular activity and tissue/organ growth at the organismal level. It can be envisioned that the dysregulation of gene expression is likely to be further exacerbated following the manifestation of the ploidy syndrome due to component scaling disproportionality and geometrical stress (Storchova et al., 2006). This vicious cycle of dysregulated gene expression and anomalous physiology/cellular structure might have led to inviability of the nonaploid plant.

It is intuitive that each species has an upper limit of ploidal level, across which the high ploidy syndrome will appear, and the severity thereof will scale with further increase of ploidal levels, but the exact ploidal thresholds may vary markedly in different species. It is also conceivable that manifestation of high ploidy syndrome depends on genome constitution of the polyploid in question. For example, octoploid *Triticale* (BBAADDRR, 2*n* = 8*x* = 56) can be readily created by crossing hexaploid wheat with diploid rye (*Secale cereale* L.) followed by chromosome doubling, and which is vigorous and fully fertile. Another relevant issue is whether the three subgenomes of hexaploid wheat are equally sensitive to multiplication. A previous study showed that induced autotetraploid of *Triticum monococcum* (A^m^A^m^) is overall smaller than their diploid counterparts although the tetraploid plants are highly fertile with normal mitosis and even show near regular meiosis (Kuspira et al., 1985). This suggests at least the A subgenome of hexaploid wheat, which is derived from *T. urartu* and is highly similar to A^m^ of *T. monococcum*, is likely sensitive to triplication due to reasons we report here. Curiously, however, we did not find that genes located to the A subgenome are particularly disturbed (as DEGs), suggesting the B and D subgenomes are probably equally sensitive to triplication. In conclusion, this study shows that triplication of the three subgenomes of hexaploid wheat causes massive disruption of overall gene expression and imposes a severely detrimental effect on growth and development at the vegetative stages. Our results, thus, suggest hexaploidy is likely the upper limit of ploidal level in wheat. We should caution, however, that the occurrence and magnitude of abnormality associated with genome triplication in wheat may also depend on genetic backgrounds and, thus, different genotypes may vary. This will remain an open question until additional nonaploid or higher ploidal level plants can be obtained in different hexaploid wheat genotypes.

## Data Availability Statement

The datasets generated for this study can be found in NCBI SRA accession PRJNA624803.

## Author Contributions

XG and BL: designed the study. XG, RL, CW, TF, and YS: analyzed the data and performed the experiments. XG, LG, HZ, and BL: summarized the data and wrote the manuscript. All authors contributed to this study, read and approved the manuscript.

## Conflict of Interest

The authors declare that the research was conducted in the absence of any commercial or financial relationships that could be construed as a potential conflict of interest.

## Footnotes

1 https://www.wheatgenome.org/

2 http://www.dnastar.com/

## Supplementary Material

FIGURE S1 Gene Ontology of nonaploid vs. hexaploid activated and silenced genes. **(A)** Cellular component. **(B)** Biological process.

FIGURE S2 Heat map of expressed syntenic triads according the FPKM values between hexaploid and nonaploid wheat. Subgenomes of both nonaploid and hexaploid were preferentially clustered together. The color key is indicated at the bottom.

FIGURE S3 The relative contribution to total gene expression by each subgenome based on triad assignment to the seven categories (detailed in Main text) between hexaploid and nonaploid wheat.

FIGURE S4 Analysis of subgenome expression partitioning by locus-specific cDNA pyrosequencing of 18 triads belonging to the balanced category and its comparison with analysis using the RNA-seq data.

TABLE S1 Details of the clean data generated in the RNA-seq data set.

TABLE S2 The list of triads used in pyrosequencing.

TABLE S3 A list of activated and silenced genes in nonaploid vs. hexaploid.
