# Supplementary figures and images for: Balanced Genome Triplication in Wheat Causes Premature Growth Arrest and an Upheaval of Genome-Wide Gene Regulation
Source: Front Genet. 2020 Jul 8;11:687. doi: 10.3389/fgene.2020.00687 (PMC7360807; doi:10.3389/fgene.2020.00687)

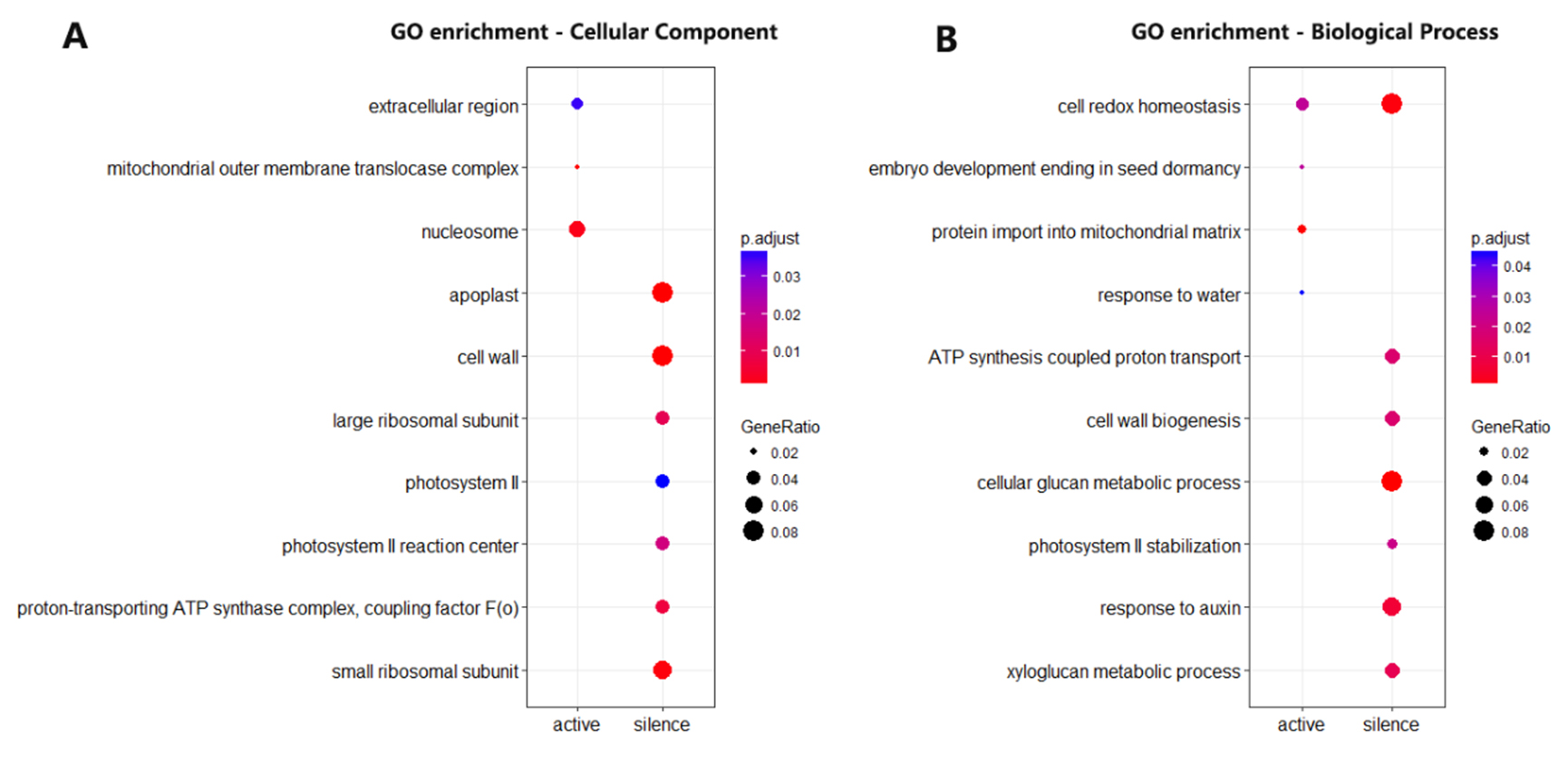

Supplement: FIGURE S1 — Gene Ontology of nonaploid vs. hexaploid activated and silenced genes. (A) Cellular component. (B) Biological process. [file Image_1.JPEG]

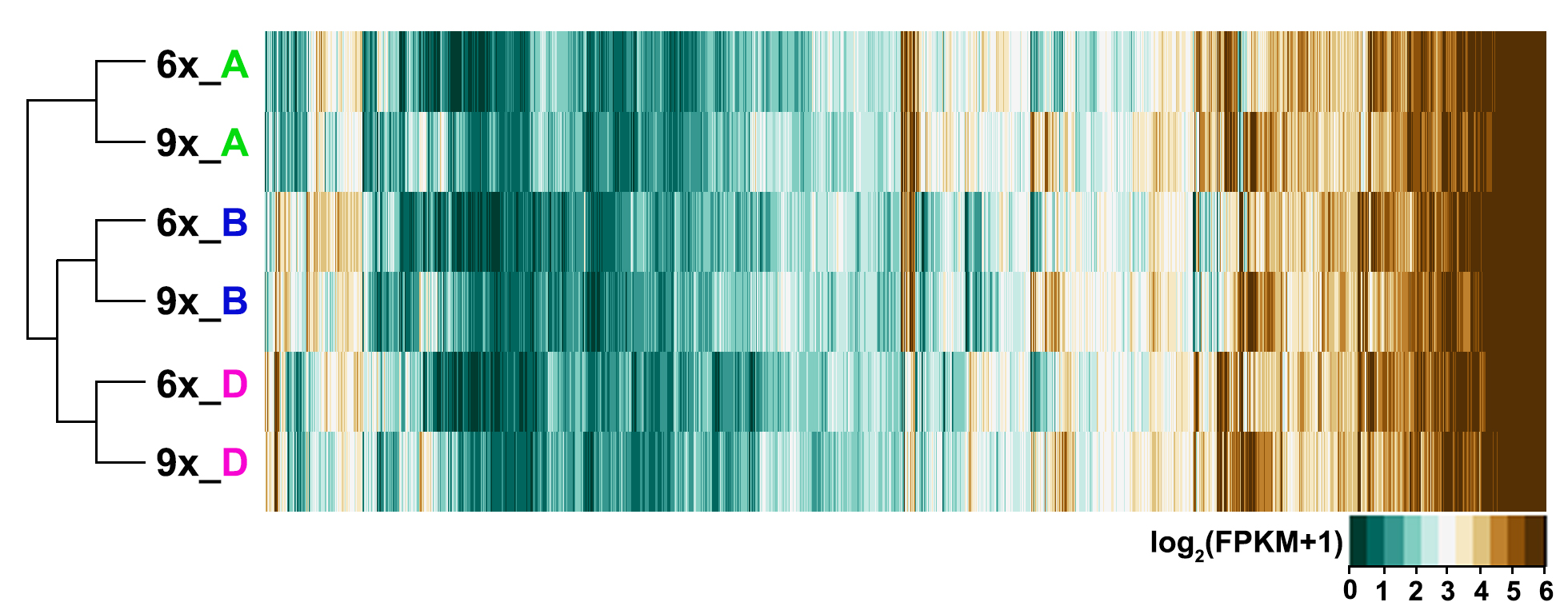

Supplement: FIGURE S2 — Heat map of expressed syntenic triads according the FPKM values between hexaploid and nonaploid wheat. Subgenomes of both nonaploid and hexaploid were preferentially clustered together. The color key is indicated at the bottom. [file Image_2.JPEG]

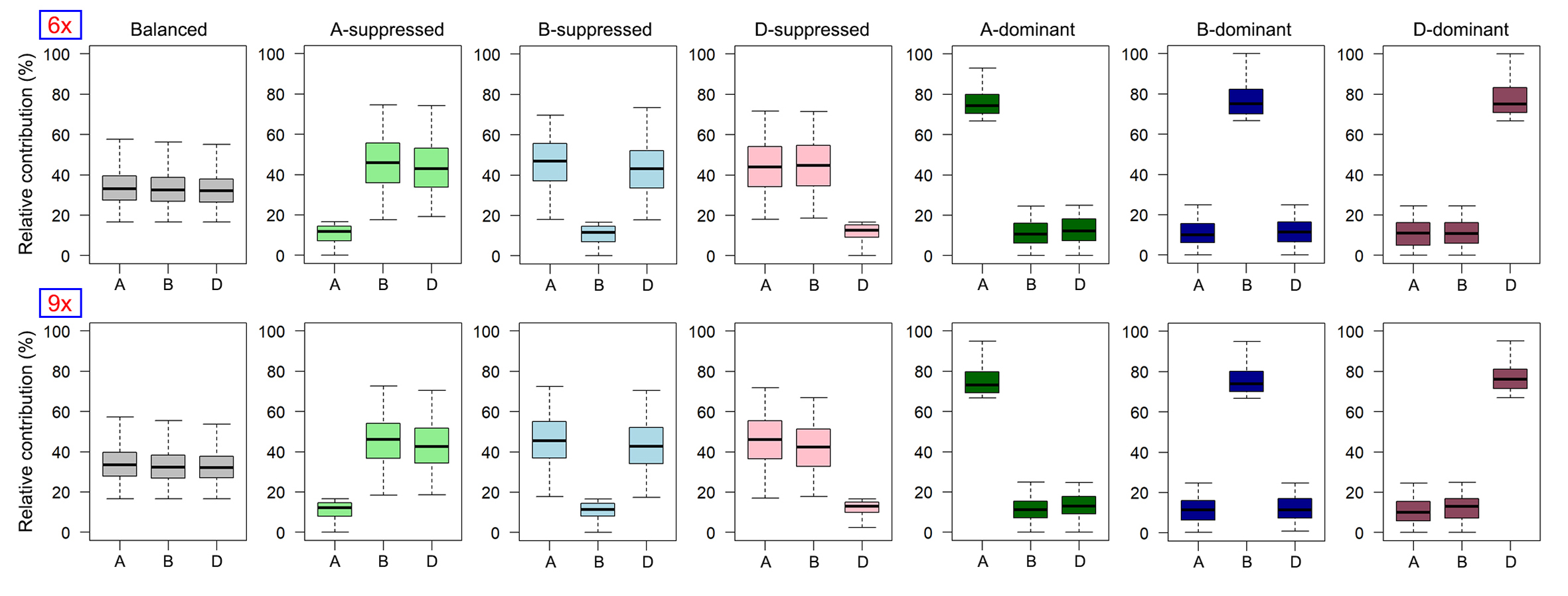

Supplement: FIGURE S3 — The relative contribution to total gene expression by each subgenome based on triad assignment to the seven categories (detailed in Main text) between hexaploid and nonaploid wheat. [file Image_3.JPEG]

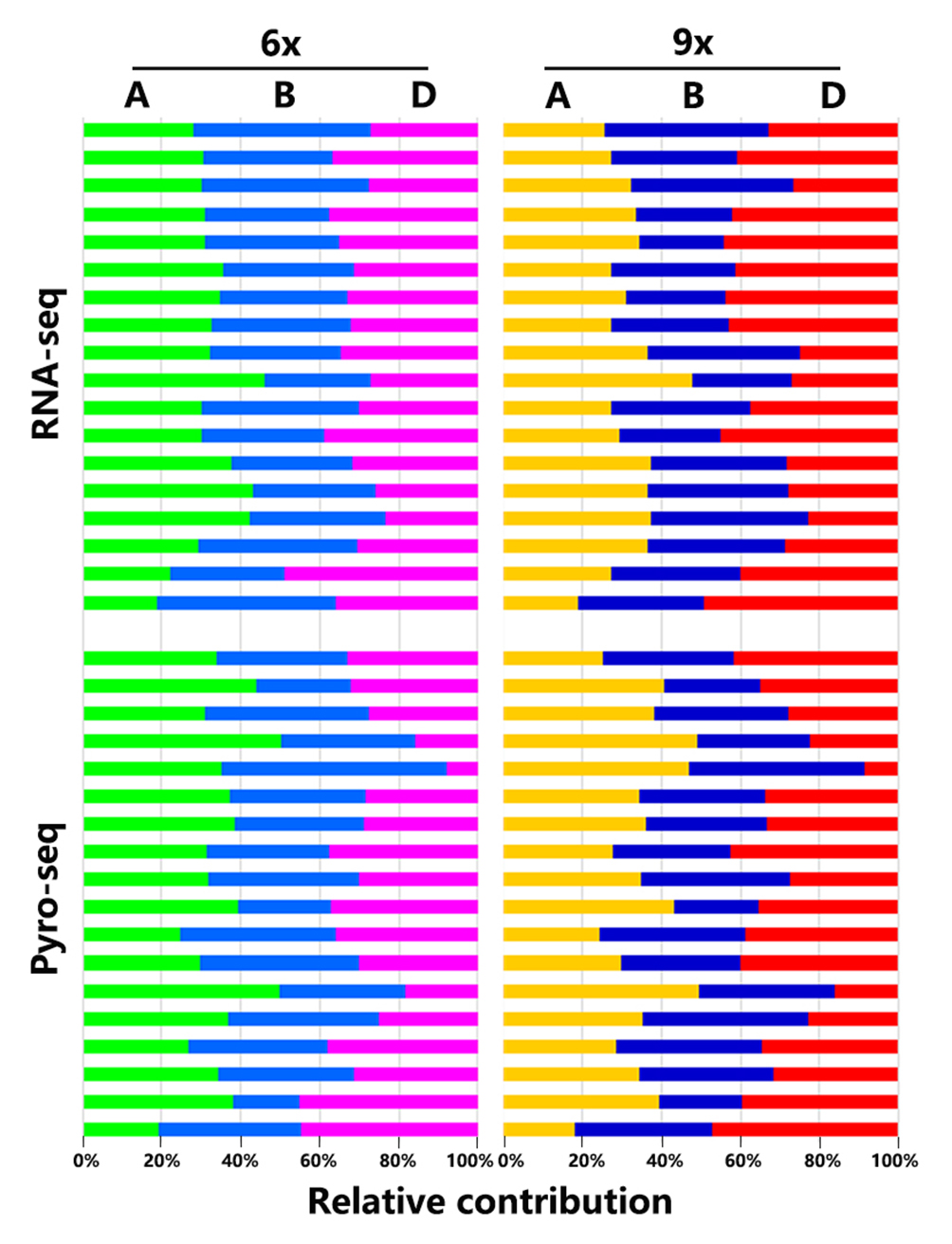

Supplement: FIGURE S4 — Analysis of subgenome expression partitioning by locus-specific cDNA pyrosequencing of 18 triads belonging to the balanced category and its comparison with analysis using the RNA-seq data. [file Image_4.JPEG]
