# Supplementary material for: Balanced Genome Triplication in Wheat Causes Premature Growth Arrest and an Upheaval of Genome-Wide Gene Regulation
Source: Front Genet. 2020 Jul 8;11:687. doi: 10.3389/fgene.2020.00687 (PMC7360807; doi:10.3389/fgene.2020.00687)
Supplement: TABLE S1 — Details of the clean data generated in the RNA-seq data set. [file Table_1.DOCX]

**Supplementary Table S1** Details of the clean data generated in the RNA-seq data set.

| **Sample** | **Replication** | **Total Reads** | **Mapped Reads** | **Mapped Rate (%)** | **Correlation Coefficient** |
| --- | --- | --- | --- | --- | --- |
|  | 1 | 188794671 | 174792162 | 92.58 |  |
| **6x** | 2 | 193183859 | 178657136 | 92.48 | 0.96 |
|  | 3 | 265570295 | 246670295 | 92.89 |  |
|  | 1 | 114807294 | 102767050 | 89.51 |  |
| **9x** | 2 | 115966444 | 100617221 | 86.76 | 0.98 |
|  | 3 | 109195978 | 94911864 | 86.92 |  |

**Supplementary Table S2** The list of triads used in pyrosequencing.

| **No.** | **A-subgenome** | **B-subgenome** | **D-subgenome** |
| --- | --- | --- | --- |
| 1 | TraesCS1A01G067900 | TraesCS1B01G086100 | TraesCS1D01G069300 |
| 2 | TraesCS1A01G112500 | TraesCS1B01G132700 | TraesCS1D01G114100 |
| 3 | TraesCS2A01G128400 | TraesCS2B01G150500 | TraesCS2D01G130900 |
| 4 | TraesCS2A01G281700 | TraesCS2B01G299000 | TraesCS2D01G280600 |
| 5 | TraesCS2A01G284700 | TraesCS2B01G301800 | TraesCS2D01G283400 |
| 6 | TraesCS2A01G288300 | TraesCS2B01G305100 | TraesCS2D01G286600 |
| 7 | TraesCS4A01G063800 | TraesCS4B01G240900 | TraesCS4D01G240700 |
| 8 | TraesCS4A01G089800 | TraesCS4B01G214500 | TraesCS4D01G215100 |
| 9 | TraesCS4A01G201200 | TraesCS4B01G105300 | TraesCS4D01G102200 |
| 10 | TraesCS4A01G297300 | TraesCS4B01G016400 | TraesCS4D01G014800 |
| 11 | TraesCS5A01G015600 | TraesCS5B01G013900 | TraesCS5D01G021600 |
| 12 | TraesCS5A01G115100 | TraesCS5B01G116100 | TraesCS5D01G125700 |
| 13 | TraesCS5A01G502700 | TraesCS4B01G331300 | TraesCS4D01G328200 |
| 14 | TraesCS6A01G090300 | TraesCS6B01G112400 | TraesCS6D01G078000 |
| 15 | TraesCS6A01G097100 | TraesCS6B01G125600 | TraesCS6D01G085300 |
| 16 | TraesCS6A01G144900 | TraesCS6B01G173100 | TraesCS6D01G133900 |
| 17 | TraesCS7A01G158900 | TraesCS7B01G063400 | TraesCS7D01G159800 |
| 18 | TraesCS7A01G196300 | TraesCS7B01G102000 | TraesCS7D01G197800 |
